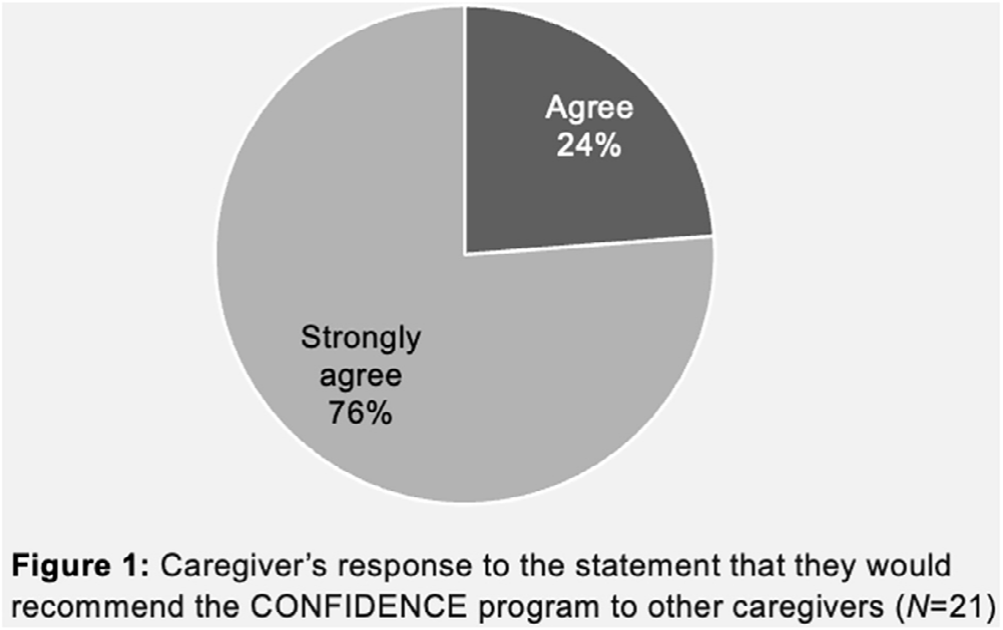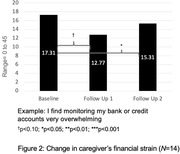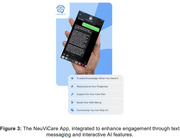# Feasibility and refinement of a culturally tailored intervention to reduce financial strain among Latino caregivers using AI

**DOI:** 10.1002/alz.090461

**Published:** 2025-01-09

**Authors:** Kylie Meyer, Chitra Dorai, Jaime Perales, Frank Puga, Jaclene A Zauszniewski, Donna Marie Benton

**Affiliations:** ^1^ Case Western, Cleveland, OH USA; ^2^ Amicus Brain Innovations, Inc., Chappaqua, NY USA; ^3^ University of Kansas Alzheimer’s Disease Center, Fairway, KS USA; ^4^ University of Kansas Alzheimer’s Disease Research Center, Fairway, KS USA; ^5^ The University of Alabama at Birmingham, Birmingham, AL USA; ^6^ Case Western Reserve University, Cleveland, OH USA; ^7^ University of Southern California, Los Angeles, OH USA

## Abstract

**Background:**

Family caregivers to persons living with dementia are at risk of financial strain from the high out‐of‐pocket costs of care and reduced employment opportunities. Financial strain disproportionately affects U.S. Latino caregivers, who experience higher care costs proportionate to household incomes than non‐Latinos. In 2022, the authors presented the preliminary results of a pilot study to test a culturally tailored psychoeducational intervention (CONFIDENCE) to reduce financial strain; we summarize the results of this study and describe planned intervention refinements.

**Method:**

We conducted a pilot study to test the feasibility of the CONFIDENCE intervention from May 2021 to September 2023. CONFIDENCE is a 5‐week group intervention delivered by Zoom by a community‐based organization in Los Angeles, CA. Caregivers could register to participate in CONFIDENCE, and, if eligible, enroll in an optional pre‐ and post‐test trial. Those in the trial completed a self‐administered online survey before the first session, post‐intervention, and 2‐ months post‐intervention. Surveys measured financial strain, and anticipated mechanisms (resourcefulness and self‐efficacy). Scores were compared using paired t‐tests.

**Result:**

136 caregivers registered to attend CONFIDENCE, among whom 69 attended at least one session (51%). On average, the 69 attendees participated in 3.13 of 5 sessions (SD = 0.17). The 21 attendees who completed the satisfaction survey indicated they would recommend CONFIDENCE (Figure 1). Moreover, the 20 caregivers who enrolled in the optional trial reported lower levels of financial strain after participating in CONFIDENCE (p‐value = 0.013), as well as increased resourcefulness and self‐efficacy two months post‐intervention (*p*‐value = 0.015 and <0.001, respectively; Figure 2).

**Conclusion:**

Assessed with Bowen et al.’s (2009) feasibility model, CONFIDENCE demonstrated high acceptability, adequate demand from end‐users, preliminary efficacy, and the ability to be implemented in a community setting. However, low attendance suggests the intervention may not be practical for end‐users to attend. CONFIDENCE is being modified to improve feasibility by consolidating sessions from 5 to 4 and integrating a digital application. This app, provided in partnership with Amicus Brian Innovations, includes text message reminders and access to interactive AI digital assistance (Figure 3). A second pilot is being conducted to examine the ongoing feasibility of the refined intervention.